# Supplementary material for: Autonomic responses to aerobic and resistance exercise in patients with chronic musculoskeletal pain: A systematic review
Source: PLoS One. 2023 Aug 14;18(8):e0290061. doi: 10.1371/journal.pone.0290061 (PMC10424875; doi:10.1371/journal.pone.0290061)
Supplement: S3 Table — (PDF) [file pone.0290061.s003.pdf]

No 1. Cordero (1996) Total score:10 (poor)

Scale	Subscale	Y(1)	U(0)	N(0)
**Reporting**	1. Is the hypothesis/aim/objective of the study clearly described?	✓		
	2. Are the main outcomes to be measured clearly described in the Introduction or Methods section?	✓		
	3. Are the characteristics of the patients included in the study clearly described?	✓		
	4. Are the interventions of interest clearly described?			✓
	5. Are the distributions of principal confounders in each group of subjects to be compared clearly described?		✓	
	6. Are the main findings of the study clearly described?	✓		
	7. Does the study provide estimates of the random variability in the data for the main outcomes?	✓		
	8. Have all important adverse events that may be a consequence of the intervention been reported?			✓
	9. Have the characteristics of patients lost to follow-up been described?			✓
	10. Have actual probability values been reported (e.g. 0.035 rather than <0.05) for the main outcomes except where the probability value is less than 0.001?			✓
**External**	11. Were the subjects asked to participate in the study representative of the entire population from which they were recruited?			✓
	12. Were those subjects who were prepared to participate representative of the entire population from which they were recruited?			✓
	13. Were the staff, places, and facilities where the patients were treated, representative of the treatment the majority of patients receive?		✓	
**Internal validity**	14. Was an attempt made to blind study subjects to the intervention they have received?			✓
	15. Was an attempt made to blind those measuring the main outcomes of the intervention?		✓	
	16. If any of the results of the study were based on "data dredging", was this made clear?		✓	
	17. In trials and cohort studies, do the analyses adjust for different lengths of follow-up of patients, or in case-control studies, is the time period between the intervention and outcome the same for cases and controls?	✓		
	18. Were the statistical tests used to assess the main outcomes appropriate?	✓		
	19. Was compliance with the intervention/s reliable?	✓		
	20. Were the main outcome measures used accurate (valid and reliable)?		✓	
**Internal validity confounding**	21. Were the patients in different intervention groups (trials and cohort studies) or were the cases and controls (case-control studies) recruited from the same population?		✓	
	22. Were study subjects in different intervention groups (trials and cohort studies) or were the cases and controls (case-control studies) recruited over the same period of time?		✓	
	23. Were study subjects randomised to intervention groups?			✓
	24. Was the randomised intervention assignment concealed from both patients and health care staff until recruitment was complete and irrevocable?			✓
	25. Was there adequate adjustment for confounding in the analyses from which the main findings were drawn?	✓		
	26. Were losses of patients to follow-up taken into account?		✓	
**Power**	27. Did the study have sufficient power to detect a clinically important effect where the probability value for a difference being due to chance is less than 5%?		✓	

No 2. da Cunha Ribeiro (2011) Total score:12 (poor)				
**Scale**	**Subscale**	**Y(1)**	**U(0)**	**N(0)**
**Reporting**	1. Is the hypothesis/aim/objective of the study clearly described?	✓		
	2. Are the main outcomes to be measured clearly described in the Introduction or Methods section?	✓		
	3. Are the characteristics of the patients included in the study clearly described?	✓		
	4. Are the interventions of interest clearly described?			✓
	5. Are the distributions of principal confounders in each group of subjects to be compared clearly described?	✓		
	6. Are the main findings of the study clearly described?	✓		
	7. Does the study provide estimates of the random variability in the data for the main outcomes?	✓		
	8. Have all important adverse events that may be a consequence of the intervention been reported?			✓
	9. Have the characteristics of patients lost to follow-up been described?			✓
	10. Have actual probability values been reported (e.g. 0.035 rather than <0.05) for the main outcomes except where the probability value is less than 0.001?	✓		
**External**	11. Were the subjects asked to participate in the study representative of the entire population from which they were recruited?			✓
	12. Were those subjects who were prepared to participate representative of the entire population from which they were recruited?			✓
	13. Were the staff, places, and facilities where the patients were treated, representative of the treatment the majority of patients receive?		✓	
**Internal validity**	14. Was an attempt made to blind study subjects to the intervention they have received?			✓
	15. Was an attempt made to blind those measuring the main outcomes of the intervention?		✓	
	16. If any of the results of the study were based on "data dredging", was this made clear?		✓	
	17. In trials and cohort studies, do the analyses adjust for different lengths of follow-up of patients, or in case-control studies, is the time period between the intervention and outcome the same for cases and controls?	✓		
	18. Were the statistical tests used to assess the main outcomes appropriate?	✓		
	19. Was compliance with the intervention/s reliable?	✓		
	20. Were the main outcome measures used accurate (valid and reliable)?		✓	
**Internal validity confounding**	21. Were the patients in different intervention groups (trials and cohort studies) or were the cases and controls (case-control studies) recruited from the same population?		✓	
	22. Were study subjects in different intervention groups (trials and cohort studies) or were the cases and controls (case-control studies) recruited over the same period of time?		✓	
	23. Were study subjects randomised to intervention groups?			✓
	24. Was the randomised intervention assignment concealed from both patients and health care staff until recruitment was complete and irrevocable?			✓
	25. Was there adequate adjustment for confounding in the analyses from which the main findings were drawn?	✓		
	26. Were losses of patients to follow-up taken into account?		✓	
**Power**	27. Did the study have sufficient power to detect a clinically important effect where the probability value for a difference being due to chance is less than 5%?		✓	

No 3. Giske (2008) Total score:17 (fair)

Scale	Subscale	Y(1)	U(0)	N(0)
**Reporting**	1. Is the hypothesis/aim/objective of the study clearly described?	✓		
	2. Are the main outcomes to be measured clearly described in the Introduction or Methods section?	✓		
	3. Are the characteristics of the patients included in the study clearly described?	✓		
	4. Are the interventions of interest clearly described?			✓
	5. Are the distributions of principal confounders in each group of subjects to be compared clearly described?		✓	
	6. Are the main findings of the study clearly described?	✓		
	7. Does the study provide estimates of the random variability in the data for the main outcomes?	✓		
	8. Have all important adverse events that may be a consequence of the intervention been reported?	✓		
	9. Have the characteristics of patients lost to follow-up been described?	✓		
	10. Have actual probability values been reported (e.g. 0.035 rather than <0.05) for the main outcomes except where the probability value is less than 0.001?	✓		
**External**	11. Were the subjects asked to participate in the study representative of the entire population from which they were recruited?	✓		
	12. Were those subjects who were prepared to participate representative of the entire population from which they were recruited?			✓
	13. Were the staff, places, and facilities where the patients were treated, representative of the treatment the majority of patients receive?		✓	
**Internal validity**	14. Was an attempt made to blind study subjects to the intervention they have received?			✓
	15. Was an attempt made to blind those measuring the main outcomes of the intervention?	✓		
	16. If any of the results of the study were based on "data dredging", was this made clear?		✓	
	17. In trials and cohort studies, do the analyses adjust for different lengths of follow-up of patients, or in case-control studies, is the time period between the intervention and outcome the same for cases and controls?	✓		
	18. Were the statistical tests used to assess the main outcomes appropriate?	✓		
	19. Was compliance with the intervention/s reliable?	✓		
	20. Were the main outcome measures used accurate (valid and reliable)?		✓	
**Internal validity confounding**	21. Were the patients in different intervention groups (trials and cohort studies) or were the cases and controls (case-control studies) recruited from the same population?		✓	
	22. Were study subjects in different intervention groups (trials and cohort studies) or were the cases and controls (case-control studies) recruited over the same period of time?		✓	
	23. Were study subjects randomised to intervention groups?			✓
	24. Was the randomised intervention assignment concealed from both patients and health care staff until recruitment was complete and irrevocable?			✓
	25. Was there adequate adjustment for confounding in the analyses from which the main findings were drawn?	✓		
	26. Were losses of patients to follow-up taken into account?	✓		
**Power**	27. Did the study have sufficient power to detect a clinically important effect where the probability value for a difference being due to chance is less than 5%?	✓		

No 4. Kadetoff (2010) Total score:13 (fair)

Scale	Subscale	Y(1)	U(0)	N(0)
**Reporting**	1. Is the hypothesis/aim/objective of the study clearly described?	✓		
	2. Are the main outcomes to be measured clearly described in the Introduction or Methods section?	✓		
	3. Are the characteristics of the patients included in the study clearly described?	✓		
	4. Are the interventions of interest clearly described?			✓
	5. Are the distributions of principal confounders in each group of subjects to be compared clearly described?		✓	
	6. Are the main findings of the study clearly described?	✓		
	7. Does the study provide estimates of the random variability in the data for the main outcomes?	✓		
	8. Have all important adverse events that may be a consequence of the intervention been reported?			✓
	9. Have the characteristics of patients lost to follow-up been described?			✓
	10. Have actual probability values been reported (e.g. 0.035 rather than <0.05) for the main outcomes except where the probability value is less than 0.001?	✓		
**External**	11. Were the subjects asked to participate in the study representative of the entire population from which they were recruited?	✓		
	12. Were those subjects who were prepared to participate representative of the entire population from which they were recruited?			✓
	13. Were the staff, places, and facilities where the patients were treated, representative of the treatment the majority of patients receive?	✓		
**Internal validity**	14. Was an attempt made to blind study subjects to the intervention they have received?			✓
	15. Was an attempt made to blind those measuring the main outcomes of the intervention?		✓	
	16. If any of the results of the study were based on "data dredging", was this made clear?		✓	
	17. In trials and cohort studies, do the analyses adjust for different lengths of follow-up of patients, or in case-control studies, is the time period between the intervention and outcome the same for cases and controls?	✓		
	18. Were the statistical tests used to assess the main outcomes appropriate?	✓		
	19. Was compliance with the intervention/s reliable?	✓		
	20. Were the main outcome measures used accurate (valid and reliable)?		✓	
**Internal validity confounding**	21. Were the patients in different intervention groups (trials and cohort studies) or were the cases and controls (case-control studies) recruited from the same population?		✓	
	22. Were study subjects in different intervention groups (trials and cohort studies) or were the cases and controls (case-control studies) recruited over the same period of time?		✓	
	23. Were study subjects randomised to intervention groups?			✓
	24. Was the randomised intervention assignment concealed from both patients and health care staff until recruitment was complete and irrevocable?			✓
	25. Was there adequate adjustment for confounding in the analyses from which the main findings were drawn?	✓		
	26. Were losses of patients to follow-up taken into account?		✓	
**Power**	27. Did the study have sufficient power to detect a clinically important effect where the probability value for a difference being due to chance is less than 5%?		✓	

No 5. Kadetoff (2007) Total score:13 (fair)				
**Scale**	**Subscale**	**Y(1)**	**U(0)**	**N(0)**
**Reporting**	1. Is the hypothesis/aim/objective of the study clearly described?	✓		
	2. Are the main outcomes to be measured clearly described in the Introduction or Methods section?	✓		
	3. Are the characteristics of the patients included in the study clearly described?	✓		
	4. Are the interventions of interest clearly described?			✓
	5. Are the distributions of principal confounders in each group of subjects to be compared clearly described?		✓	
	6. Are the main findings of the study clearly described?	✓		
	7. Does the study provide estimates of the random variability in the data for the main outcomes?	✓		
	8. Have all important adverse events that may be a consequence of the intervention been reported?			✓
	9. Have the characteristics of patients lost to follow-up been described?			✓
	10. Have actual probability values been reported (e.g. 0.035 rather than <0.05) for the main outcomes except where the probability value is less than 0.001?	✓		
**External**	11. Were the subjects asked to participate in the study representative of the entire population from which they were recruited?	✓		
	12. Were those subjects who were prepared to participate representative of the entire population from which they were recruited?			✓
	13. Were the staff, places, and facilities where the patients were treated, representative of the treatment the majority of patients receive?	✓		
**Internal validity**	14. Was an attempt made to blind study subjects to the intervention they have received?			✓
	15. Was an attempt made to blind those measuring the main outcomes of the intervention?			✓
	16. If any of the results of the study were based on "data dredging", was this made clear?		✓	
	17. In trials and cohort studies, do the analyses adjust for different lengths of follow-up of patients, or in case-control studies, is the time period between the intervention and outcome the same for cases and controls?	✓		
	18. Were the statistical tests used to assess the main outcomes appropriate?	✓		
	19. Was compliance with the intervention/s reliable?	✓		
	20. Were the main outcome measures used accurate (valid and reliable)?		✓	
**Internal validity confounding**	21. Were the patients in different intervention groups (trials and cohort studies) or were the cases and controls (case-control studies) recruited from the same population?		✓	
	22. Were study subjects in different intervention groups (trials and cohort studies) or were the cases and controls (case-control studies) recruited over the same period of time?		✓	
	23. Were study subjects randomised to intervention groups?			✓
	24. Was the randomised intervention assignment concealed from both patients and health care staff until recruitment was complete and irrevocable?			✓
	25. Was there adequate adjustment for confounding in the analyses from which the main findings were drawn?	✓		
	26. Were losses of patients to follow-up taken into account?		✓	
**Power**	27. Did the study have sufficient power to detect a clinically important effect where the probability value for a difference being due to chance is less than 5%?		✓	

No 6. Kaya (2010) Total score:13 (fair)				
**Scale**	**Subscale**	**Y(1)**	**U(0)**	**N(0)**
**Reporting**	1. Is the hypothesis/aim/objective of the study clearly described?	✓		
	2. Are the main outcomes to be measured clearly described in the Introduction or Methods section?	✓		
	3. Are the characteristics of the patients included in the study clearly described?	✓		
	4. Are the interventions of interest clearly described?			✓
	5. Are the distributions of principal confounders in each group of subjects to be compared clearly described?	✓		
	6. Are the main findings of the study clearly described?	✓		
	7. Does the study provide estimates of the random variability in the data for the main outcomes?	✓		
	8. Have all important adverse events that may be a consequence of the intervention been reported?	✓		
	9. Have the characteristics of patients lost to follow-up been described?			✓
	10. Have actual probability values been reported (e.g. 0.035 rather than <0.05) for the main outcomes except where the probability value is less than 0.001?	✓		
**External**	11. Were the subjects asked to participate in the study representative of the entire population from which they were recruited?		✓	
	12. Were those subjects who were prepared to participate representative of the entire population from which they were recruited?			✓
	13. Were the staff, places, and facilities where the patients were treated, representative of the treatment the majority of patients receive?		✓	
**Internal validity**	14. Was an attempt made to blind study subjects to the intervention they have received?			✓
	15. Was an attempt made to blind those measuring the main outcomes of the intervention?		✓	
	16. If any of the results of the study were based on "data dredging", was this made clear?		✓	
	17. In trials and cohort studies, do the analyses adjust for different lengths of follow-up of patients, or in case-control studies, is the time period between the intervention and outcome the same for cases and controls?	✓		
	18. Were the statistical tests used to assess the main outcomes appropriate?	✓		
	19. Was compliance with the intervention/s reliable?	✓		
	20. Were the main outcome measures used accurate (valid and reliable)?		✓	
**Internal validity confounding**	21. Were the patients in different intervention groups (trials and cohort studies) or were the cases and controls (case-control studies) recruited from the same population?		✓	
	22. Were study subjects in different intervention groups (trials and cohort studies) or were the cases and controls (case-control studies) recruited over the same period of time?		✓	
	23. Were study subjects randomised to intervention groups?			✓
	24. Was the randomised intervention assignment concealed from both patients and health care staff until recruitment was complete and irrevocable?			✓
	25. Was there adequate adjustment for confounding in the analyses from which the main findings were drawn?	✓		
	26. Were losses of patients to follow-up taken into account?		✓	
**Power**	27. Did the study have sufficient power to detect a clinically important effect where the probability value for a difference being due to chance is less than 5%?		✓	

No 7. Kingsley (2009) Total score:13 (fair)

Scale	Subscale	Y(1)	U(0)	N(0)
**Reporting**	1. Is the hypothesis/aim/objective of the study clearly described?	✓		
	2. Are the main outcomes to be measured clearly described in the Introduction or Methods section?	✓		
	3. Are the characteristics of the patients included in the study clearly described?	✓		
	4. Are the interventions of interest clearly described?			✓
	5. Are the distributions of principal confounders in each group of subjects to be compared clearly described?	✓		
	6. Are the main findings of the study clearly described?	✓		
	7. Does the study provide estimates of the random variability in the data for the main outcomes?	✓		
	8. Have all important adverse events that may be a consequence of the intervention been reported?			✓
	9. Have the characteristics of patients lost to follow-up been described?			✓
	10. Have actual probability values been reported (e.g. 0.035 rather than <0.05) for the main outcomes except where the probability value is less than 0.001?	✓		
**External**	11. Were the subjects asked to participate in the study representative of the entire population from which they were recruited?	✓		
	12. Were those subjects who were prepared to participate representative of the entire population from which they were recruited?			✓
	13. Were the staff, places, and facilities where the patients were treated, representative of the treatment the majority of patients receive?			✓
**Internal validity**	14. Was an attempt made to blind study subjects to the intervention they have received?			✓
	15. Was an attempt made to blind those measuring the main outcomes of the intervention?		✓	
	16. If any of the results of the study were based on "data dredging", was this made clear?		✓	
	17. In trials and cohort studies, do the analyses adjust for different lengths of follow-up of patients, or in case-control studies, is the time period between the intervention and outcome the same for cases and controls?	✓		
	18. Were the statistical tests used to assess the main outcomes appropriate?	✓		
	19. Was compliance with the intervention/s reliable?	✓		
	20. Were the main outcome measures used accurate (valid and reliable)?		✓	
**Internal validity confounding**	21. Were the patients in different intervention groups (trials and cohort studies) or were the cases and controls (case-control studies) recruited from the same population?		✓	
	22. Were study subjects in different intervention groups (trials and cohort studies) or were the cases and controls (case-control studies) recruited over the same period of time?		✓	
	23. Were study subjects randomised to intervention groups?			✓
	24. Was the randomised intervention assignment concealed from both patients and health care staff until recruitment was complete and irrevocable?			✓
	25. Was there adequate adjustment for confounding in the analyses from which the main findings were drawn?	✓		
	26. Were losses of patients to follow-up taken into account?		✓	
**Power**	27. Did the study have sufficient power to detect a clinically important effect where the probability value for a difference being due to chance is less than 5%?		✓	

No 8. Maia (2016) Total score:17 (fair)				
**Scale**	**Subscale**	**Y(1)**	**U(0)**	**N(0)**
**Reporting**	1. Is the hypothesis/aim/objective of the study clearly described?	✓		
	2. Are the main outcomes to be measured clearly described in the Introduction or Methods section?	✓		
	3. Are the characteristics of the patients included in the study clearly described?	✓		
	4. Are the interventions of interest clearly described?			✓
	5. Are the distributions of principal confounders in each group of subjects to be compared clearly described?	✓		
	6. Are the main findings of the study clearly described?	✓		
	7. Does the study provide estimates of the random variability in the data for the main outcomes?	✓		
	8. Have all important adverse events that may be a consequence of the intervention been reported?			✓
	9. Have the characteristics of patients lost to follow-up been described?			✓
	10. Have actual probability values been reported (e.g. 0.035 rather than <0.05) for the main outcomes except where the probability value is less than 0.001?	✓		
**External**	11. Were the subjects asked to participate in the study representative of the entire population from which they were recruited?	✓		
	12. Were those subjects who were prepared to participate representative of the entire population from which they were recruited?	✓		
	13. Were the staff, places, and facilities where the patients were treated, representative of the treatment the majority of patients receive?	✓		
**Internal validity**	14. Was an attempt made to blind study subjects to the intervention they have received?			✓
	15. Was an attempt made to blind those measuring the main outcomes of the intervention?	✓		
	16. If any of the results of the study were based on "data dredging", was this made clear?		✓	
	17. In trials and cohort studies, do the analyses adjust for different lengths of follow-up of patients, or in case-control studies, is the time period between the intervention and outcome the same for cases and controls?	✓		
	18. Were the statistical tests used to assess the main outcomes appropriate?	✓		
	19. Was compliance with the intervention/s reliable?	✓		
	20. Were the main outcome measures used accurate (valid and reliable)?		✓	
**Internal validity confounding**	21. Were the patients in different intervention groups (trials and cohort studies) or were the cases and controls (case-control studies) recruited from the same population?		✓	
	22. Were study subjects in different intervention groups (trials and cohort studies) or were the cases and controls (case-control studies) recruited over the same period of time?	✓		
	23. Were study subjects randomised to intervention groups?			✓
	24. Was the randomised intervention assignment concealed from both patients and health care staff until recruitment was complete and irrevocable?			✓
	25. Was there adequate adjustment for confounding in the analyses from which the main findings were drawn?	✓		
	26. Were losses of patients to follow-up taken into account?		✓	
**Power**	27. Did the study have sufficient power to detect a clinically important effect where the probability value for a difference being due to chance is less than 5%?		✓	

No 9. Oosterwijck (2017) Total score:18 (fair)

Scale	Subscale	Y(1)	U(0)	N(0)
**Reporting**	1. Is the hypothesis/aim/objective of the study clearly described?	✓		
	2. Are the main outcomes to be measured clearly described in the Introduction or Methods section?	✓		
	3. Are the characteristics of the patients included in the study clearly described?	✓		
	4. Are the interventions of interest clearly described?			✓
	5. Are the distributions of principal confounders in each group of subjects to be compared clearly described?	✓		
	6. Are the main findings of the study clearly described?	✓		
	7. Does the study provide estimates of the random variability in the data for the main outcomes?	✓		
	8. Have all important adverse events that may be a consequence of the intervention been reported?			✓
	9. Have the characteristics of patients lost to follow-up been described?	✓		
	10. Have actual probability values been reported (e.g. 0.035 rather than <0.05) for the main outcomes except where the probability value is less than 0.001?	✓		
**External**	11. Were the subjects asked to participate in the study representative of the entire population from which they were recruited?	✓		
	12. Were those subjects who were prepared to participate representative of the entire population from which they were recruited?			✓
	13. Were the staff, places, and facilities where the patients were treated, representative of the treatment the majority of patients receive?			✓
**Internal validity**	14. Was an attempt made to blind study subjects to the intervention they have received?			✓
	15. Was an attempt made to blind those measuring the main outcomes of the intervention?		✓	
	16. If any of the results of the study were based on "data dredging", was this made clear?	✓		
	17. In trials and cohort studies, do the analyses adjust for different lengths of follow-up of patients, or in case-control studies, is the time period between the intervention and outcome the same for cases and controls?	✓		
	18. Were the statistical tests used to assess the main outcomes appropriate?	✓		
	19. Was compliance with the intervention/s reliable?	✓		
	20. Were the main outcome measures used accurate (valid and reliable)?	✓		
**Internal validity confounding**	21. Were the patients in different intervention groups (trials and cohort studies) or were the cases and controls (case-control studies) recruited from the same population?		✓	
	22. Were study subjects in different intervention groups (trials and cohort studies) or were the cases and controls (case-control studies) recruited over the same period of time?		✓	
	23. Were study subjects randomised to intervention groups?			✓
	24. Was the randomised intervention assignment concealed from both patients and health care staff until recruitment was complete and irrevocable?			✓
	25. Was there adequate adjustment for confounding in the analyses from which the main findings were drawn?	✓		
	26. Were losses of patients to follow-up taken into account?	✓		
**Power**	27. Did the study have sufficient power to detect a clinically important effect where the probability value for a difference being due to chance is less than 5%?	✓		

No 10. Peçanha (2021) Total score:19 (good)

Scale	Subscale	Y(1)	U(0)	N(0)
**Reporting**	1. Is the hypothesis/aim/objective of the study clearly described?	✓		
	2. Are the main outcomes to be measured clearly described in the Introduction or Methods section?	✓		
	3. Are the characteristics of the patients included in the study clearly described?	✓		
	4. Are the interventions of interest clearly described?			✓
	5. Are the distributions of principal confounders in each group of subjects to be compared clearly described?	✓		
	6. Are the main findings of the study clearly described?	✓		
	7. Does the study provide estimates of the random variability in the data for the main outcomes?	✓		
	8. Have all important adverse events that may be a consequence of the intervention been reported?	✓		
	9. Have the characteristics of patients lost to follow-up been described?	✓		
	10. Have actual probability values been reported (e.g. 0.035 rather than <0.05) for the main outcomes except where the probability value is less than 0.001?	✓		
**External**	11. Were the subjects asked to participate in the study representative of the entire population from which they were recruited?	✓		
	12. Were those subjects who were prepared to participate representative of the entire population from which they were recruited?			✓
	13. Were the staff, places, and facilities where the patients were treated, representative of the treatment the majority of patients receive?	✓		
**Internal validity**	14. Was an attempt made to blind study subjects to the intervention they have received?			✓
	15. Was an attempt made to blind those measuring the main outcomes of the intervention?		✓	
	16. If any of the results of the study were based on "data dredging", was this made clear?	✓		
	17. In trials and cohort studies, do the analyses adjust for different lengths of follow-up of patients, or in case-control studies, is the time period between the intervention and outcome the same for cases and controls?	✓		
	18. Were the statistical tests used to assess the main outcomes appropriate?	✓		
	19. Was compliance with the intervention/s reliable?	✓		
	20. Were the main outcome measures used accurate (valid and reliable)?		✓	
**Internal validity confounding**	21. Were the patients in different intervention groups (trials and cohort studies) or were the cases and controls (case-control studies) recruited from the same population?		✓	
	22. Were study subjects in different intervention groups (trials and cohort studies) or were the cases and controls (case-control studies) recruited over the same period of time?	✓		
	23. Were study subjects randomised to intervention groups?			✓
	24. Was the randomised intervention assignment concealed from both patients and health care staff until recruitment was complete and irrevocable?			✓
	25. Was there adequate adjustment for confounding in the analyses from which the main findings were drawn?	✓		
	26. Were losses of patients to follow-up taken into account?	✓		
**Power**	27. Did the study have sufficient power to detect a clinically important effect where the probability value for a difference being due to chance is less than 5%?		✓	

**No 11. Peçanha (2018) Total score:16 (fair)**				
**Scale**	**Subscale**	**Y(1)**	**U(0)**	**N(0)**
**Reporting**	1. Is the hypothesis/aim/objective of the study clearly described?	✓		
	2. Are the main outcomes to be measured clearly described in the Introduction or Methods section?	✓		
	3. Are the characteristics of the patients included in the study clearly described?	✓		
	4. Are the interventions of interest clearly described?			✓
	5. Are the distributions of principal confounders in each group of subjects to be compared clearly described?	✓		
	6. Are the main findings of the study clearly described?	✓		
	7. Does the study provide estimates of the random variability in the data for the main outcomes?	✓		
	8. Have all important adverse events that may be a consequence of the intervention been reported?			✓
	9. Have the characteristics of patients lost to follow-up been described?	✓		
	10. Have actual probability values been reported (e.g. 0.035 rather than <0.05) for the main outcomes except where the probability value is less than 0.001?	✓		
**External**	11. Were the subjects asked to participate in the study representative of the entire population from which they were recruited?	✓		
	12. Were those subjects who were prepared to participate representative of the entire population from which they were recruited?			✓
	13. Were the staff, places, and facilities where the patients were treated, representative of the treatment the majority of patients receive?	✓		
**Internal validity**	14. Was an attempt made to blind study subjects to the intervention they have received?			✓
	15. Was an attempt made to blind those measuring the main outcomes of the intervention?		✓	
	16. If any of the results of the study were based on "data dredging", was this made clear?		✓	
	17. In trials and cohort studies, do the analyses adjust for different lengths of follow-up of patients, or in case-control studies, is the time period between the intervention and outcome the same for cases and controls?	✓		
	18. Were the statistical tests used to assess the main outcomes appropriate?	✓		
	19. Was compliance with the intervention/s reliable?	✓		
	20. Were the main outcome measures used accurate (valid and reliable)?		✓	
**Internal validity confounding**	21. Were the patients in different intervention groups (trials and cohort studies) or were the cases and controls (case-control studies) recruited from the same population?		✓	
	22. Were study subjects in different intervention groups (trials and cohort studies) or were the cases and controls (case-control studies) recruited over the same period of time?		✓	
	23. Were study subjects randomised to intervention groups?			✓
	24. Was the randomised intervention assignment concealed from both patients and health care staff until recruitment was complete and irrevocable?			✓
	25. Was there adequate adjustment for confounding in the analyses from which the main findings were drawn?	✓		
	26. Were losses of patients to follow-up taken into account?	✓		
**Power**	27. Did the study have sufficient power to detect a clinically important effect where the probability value for a difference being due to chance is less than 5%?		✓	

No 12. Shiro (2012) Total score:12 (poor)

Scale	Subscale	Y(1)	U(0)	N(0)
**Reporting**	1. Is the hypothesis/aim/objective of the study clearly described?	✓		
	2. Are the main outcomes to be measured clearly described in the Introduction or Methods section?	✓		
	3. Are the characteristics of the patients included in the study clearly described?	✓		
	4. Are the interventions of interest clearly described?			✓
	5. Are the distributions of principal confounders in each group of subjects to be compared clearly described?	✓		
	6. Are the main findings of the study clearly described?	✓		
	7. Does the study provide estimates of the random variability in the data for the main outcomes?	✓		
	8. Have all important adverse events that may be a consequence of the intervention been reported?			✓
	9. Have the characteristics of patients lost to follow-up been described?			✓
	10. Have actual probability values been reported (e.g. 0.035 rather than <0.05) for the main outcomes except where the probability value is less than 0.001?	✓		
**External**	11. Were the subjects asked to participate in the study representative of the entire population from which they were recruited?			✓
	12. Were those subjects who were prepared to participate representative of the entire population from which they were recruited?			✓
	13. Were the staff, places, and facilities where the patients were treated, representative of the treatment the majority of patients receive?		✓	
**Internal validity**	14. Was an attempt made to blind study subjects to the intervention they have received?			✓
	15. Was an attempt made to blind those measuring the main outcomes of the intervention?		✓	
	16. If any of the results of the study were based on "data dredging", was this made clear?		✓	
	17. In trials and cohort studies, do the analyses adjust for different lengths of follow-up of patients, or in case-control studies, is the time period between the intervention and outcome the same for cases and controls?	✓		
	18. Were the statistical tests used to assess the main outcomes appropriate?	✓		
	19. Was compliance with the intervention/s reliable?	✓		
	20. Were the main outcome measures used accurate (valid and reliable)?		✓	
**Internal validity confounding**	21. Were the patients in different intervention groups (trials and cohort studies) or were the cases and controls (case-control studies) recruited from the same population?		✓	
	22. Were study subjects in different intervention groups (trials and cohort studies) or were the cases and controls (case-control studies) recruited over the same period of time?		✓	
	23. Were study subjects randomised to intervention groups?			✓
	24. Was the randomised intervention assignment concealed from both patients and health care staff until recruitment was complete and irrevocable?			✓
	25. Was there adequate adjustment for confounding in the analyses from which the main findings were drawn?	✓		
	26. Were losses of patients to follow-up taken into account?		✓	
**Power**	27. Did the study have sufficient power to detect a clinically important effect where the probability value for a difference being due to chance is less than 5%?		✓	

No 13. Bardal (2015) Total score:18 (fair)

Scale	Subscale	Y(1)	U(0)	N(0)
**Reporting**	1. Is the hypothesis/aim/objective of the study clearly described?	✓		
	2. Are the main outcomes to be measured clearly described in the Introduction or Methods section?	✓		
	3. Are the characteristics of the patients included in the study clearly described?	✓		
	4. Are the interventions of interest clearly described?	✓		
	5. Are the distributions of principal confounders in each group of subjects to be compared clearly described?	✓		
	6. Are the main findings of the study clearly described?	✓		
	7. Does the study provide estimates of the random variability in the data for the main outcomes?	✓		
	8. Have all important adverse events that may be a consequence of the intervention been reported?	✓		
	9. Have the characteristics of patients lost to follow-up been described?	✓		
	10. Have actual probability values been reported (e.g. 0.035 rather than <0.05) for the main outcomes except where the probability value is less than 0.001?	✓		
**External**	11. Were the subjects asked to participate in the study representative of the entire population from which they were recruited?	✓		
	12. Were those subjects who were prepared to participate representative of the entire population from which they were recruited?	✓		
	13. Were the staff, places, and facilities where the patients were treated, representative of the treatment the majority of patients receive?		✓	
**Internal validity**	14. Was an attempt made to blind study subjects to the intervention they have received?			✓
	15. Was an attempt made to blind those measuring the main outcomes of the intervention?		✓	
	16. If any of the results of the study were based on “data dredging”, was this made clear?		✓	
	17. In trials and cohort studies, do the analyses adjust for different lengths of follow-up of patients, or in case-control studies, is the time period between the intervention and outcome the same for cases and controls?	✓		
	18. Were the statistical tests used to assess the main outcomes appropriate?	✓		
	19. Was compliance with the intervention/s reliable?	✓		
	20. Were the main outcome measures used accurate (valid and reliable)?		✓	
**Internal validity confounding**	21. Were the patients in different intervention groups (trials and cohort studies) or were the cases and controls (case-control studies) recruited from the same population?			✓
	22. Were study subjects in different intervention groups (trials and cohort studies) or were the cases and controls (case-control studies) recruited over the same period of time?		✓	
	23. Were study subjects randomised to intervention groups?			✓
	24. Was the randomised intervention assignment concealed from both patients and health care staff until recruitment was complete and irrevocable?			✓
	25. Was there adequate adjustment for confounding in the analyses from which the main findings were drawn?	✓		
	26. Were losses of patients to follow-up taken into account?	✓		
**Power**	27. Did the study have sufficient power to detect a clinically important effect where the probability value for a difference being due to chance is less than 5%?		✓	

No 14. Figueroa (2008) Total score:17 (fair)

Scale	Subscale	Y(1)	U(0)	N(0)
**Reporting**	1. Is the hypothesis/aim/objective of the study clearly described?	✓		
	2. Are the main outcomes to be measured clearly described in the Introduction or Methods section?	✓		
	3. Are the characteristics of the patients included in the study clearly described?	✓		
	4. Are the interventions of interest clearly described?	✓		
	5. Are the distributions of principal confounders in each group of subjects to be compared clearly described?	✓		
	6. Are the main findings of the study clearly described?	✓		
	7. Does the study provide estimates of the random variability in the data for the main outcomes?	✓		
	8. Have all important adverse events that may be a consequence of the intervention been reported?	✓		
	9. Have the characteristics of patients lost to follow-up been described?			✓
	10. Have actual probability values been reported (e.g. 0.035 rather than <0.05) for the main outcomes except where the probability value is less than 0.001?	✓		
**External**	11. Were the subjects asked to participate in the study representative of the entire population from which they were recruited?	✓		
	12. Were those subjects who were prepared to participate representative of the entire population from which they were recruited?			✓
	13. Were the staff, places, and facilities where the patients were treated, representative of the treatment the majority of patients receive?		✓	
**Internal validity**	14. Was an attempt made to blind study subjects to the intervention they have received?			✓
	15. Was an attempt made to blind those measuring the main outcomes of the intervention?		✓	
	16. If any of the results of the study were based on “data dredging”, was this made clear?		✓	
	17. In trials and cohort studies, do the analyses adjust for different lengths of follow-up of patients, or in case-control studies, is the time period between the intervention and outcome the same for cases and controls?	✓		
	18. Were the statistical tests used to assess the main outcomes appropriate?	✓		
	19. Was compliance with the intervention/s reliable?	✓		
	20. Were the main outcome measures used accurate (valid and reliable)?		✓	
**Internal validity confounding**	21. Were the patients in different intervention groups (trials and cohort studies) or were the cases and controls (case-control studies) recruited from the same population?	✓		
	22. Were study subjects in different intervention groups (trials and cohort studies) or were the cases and controls (case-control studies) recruited over the same period of time?		✓	
	23. Were study subjects randomised to intervention groups?			✓
	24. Was the randomised intervention assignment concealed from both patients and health care staff until recruitment was complete and irrevocable?			✓
	25. Was there adequate adjustment for confounding in the analyses from which the main findings were drawn?	✓		
	26. Were losses of patients to follow-up taken into account?	✓		
**Power**	27. Did the study have sufficient power to detect a clinically important effect where the probability value for a difference being due to chance is less than 5%?		✓	

No 15. Gavi (2014) Total score:22 (good)				
**Scale**	**Subscale**	**Y(1)**	**U(0)**	**N(0)**
**Reporting**	1. Is the hypothesis/aim/objective of the study clearly described?	✓		
	2. Are the main outcomes to be measured clearly described in the Introduction or Methods section?	✓		
	3. Are the characteristics of the patients included in the study clearly described?	✓		
	4. Are the interventions of interest clearly described?	✓		
	5. Are the distributions of principal confounders in each group of subjects to be compared clearly described?	✓		
	6. Are the main findings of the study clearly described?	✓		
	7. Does the study provide estimates of the random variability in the data for the main outcomes?	✓		
	8. Have all important adverse events that may be a consequence of the intervention been reported?			✓
	9. Have the characteristics of patients lost to follow-up been described?	✓		
	10. Have actual probability values been reported (e.g. 0.035 rather than <0.05) for the main outcomes except where the probability value is less than 0.001?	✓		
**External**	11. Were the subjects asked to participate in the study representative of the entire population from which they were recruited?	✓		
	12. Were those subjects who were prepared to participate representative of the entire population from which they were recruited?	✓		
	13. Were the staff, places, and facilities where the patients were treated, representative of the treatment the majority of patients receive?	✓		
**Internal validity**	14. Was an attempt made to blind study subjects to the intervention they have received?			✓
	15. Was an attempt made to blind those measuring the main outcomes of the intervention?	✓		
	16. If any of the results of the study were based on "data dredging", was this made clear?		✓	
	17. In trials and cohort studies, do the analyses adjust for different lengths of follow-up of patients, or in case-control studies, is the time period between the intervention and outcome the same for cases and controls?	✓		
	18. Were the statistical tests used to assess the main outcomes appropriate?	✓		
	19. Was compliance with the intervention/s reliable?	✓		
	20. Were the main outcome measures used accurate (valid and reliable)?		✓	
**Internal validity confounding**	21. Were the patients in different intervention groups (trials and cohort studies) or were the cases and controls (case-control studies) recruited from the same population?	✓		
	22. Were study subjects in different intervention groups (trials and cohort studies) or were the cases and controls (case-control studies) recruited over the same period of time?	✓		
	23. Were study subjects randomised to intervention groups?	✓		
	24. Was the randomised intervention assignment concealed from both patients and health care staff until recruitment was complete and irrevocable?			✓
	25. Was there adequate adjustment for confounding in the analyses from which the main findings were drawn?			✓
	26. Were losses of patients to follow-up taken into account?	✓		
**Power**	27. Did the study have sufficient power to detect a clinically important effect where the probability value for a difference being due to chance is less than 5%?	✓		

No 16. Kingsley (2010) Total score:19 (good)

Scale	Subscale	Y(1)	U(0)	N(0)
**Reporting**	1. Is the hypothesis/aim/objective of the study clearly described?	✓		
	2. Are the main outcomes to be measured clearly described in the Introduction or Methods section?	✓		
	3. Are the characteristics of the patients included in the study clearly described?	✓		
	4. Are the interventions of interest clearly described?	✓		
	5. Are the distributions of principal confounders in each group of subjects to be compared clearly described?	✓		
	6. Are the main findings of the study clearly described?	✓		
	7. Does the study provide estimates of the random variability in the data for the main outcomes?	✓		
	8. Have all important adverse events that may be a consequence of the intervention been reported?			✓
	9. Have the characteristics of patients lost to follow-up been described?	✓		
	10. Have actual probability values been reported (e.g. 0.035 rather than <0.05) for the main outcomes except where the probability value is less than 0.001?			✓
**External**	11. Were the subjects asked to participate in the study representative of the entire population from which they were recruited?	✓		
	12. Were those subjects who were prepared to participate representative of the entire population from which they were recruited?	✓		
	13. Were the staff, places, and facilities where the patients were treated, representative of the treatment the majority of patients receive?			✓
**Internal validity**	14. Was an attempt made to blind study subjects to the intervention they have received?			✓
	15. Was an attempt made to blind those measuring the main outcomes of the intervention?	✓		
	16. If any of the results of the study were based on "data dredging", was this made clear?		✓	
	17. In trials and cohort studies, do the analyses adjust for different lengths of follow-up of patients, or in case-control studies, is the time period between the intervention and outcome the same for cases and controls?	✓		
	18. Were the statistical tests used to assess the main outcomes appropriate?	✓		
	19. Was compliance with the intervention/s reliable?	✓		
	20. Were the main outcome measures used accurate (valid and reliable)?	✓		
**Internal validity confounding**	21. Were the patients in different intervention groups (trials and cohort studies) or were the cases and controls (case-control studies) recruited from the same population?	✓		
	22. Were study subjects in different intervention groups (trials and cohort studies) or were the cases and controls (case-control studies) recruited over the same period of time?		✓	
	23. Were study subjects randomised to intervention groups?			✓
	24. Was the randomised intervention assignment concealed from both patients and health care staff until recruitment was complete and irrevocable?			✓
	25. Was there adequate adjustment for confounding in the analyses from which the main findings were drawn?	✓		
	26. Were losses of patients to follow-up taken into account?	✓		
**Power**	27. Did the study have sufficient power to detect a clinically important effect where the probability value for a difference being due to chance is less than 5%?		✓	

No 17. Sañudo (2015) Total score:22 (good)

Scale	Subscale	Y(1)	U(0)	N(0)
**Reporting**	1. Is the hypothesis/aim/objective of the study clearly described?	✓		
	2. Are the main outcomes to be measured clearly described in the Introduction or Methods section?	✓		
	3. Are the characteristics of the patients included in the study clearly described?	✓		
	4. Are the interventions of interest clearly described?	✓		
	5. Are the distributions of principal confounders in each group of subjects to be compared clearly described?	✓		
	6. Are the main findings of the study clearly described?	✓		
	7. Does the study provide estimates of the random variability in the data for the main outcomes?	✓		
	8. Have all important adverse events that may be a consequence of the intervention been reported?			✓
	9. Have the characteristics of patients lost to follow-up been described?	✓		
	10. Have actual probability values been reported (e.g. 0.035 rather than <0.05) for the main outcomes except where the probability value is less than 0.001?	✓		
**External**	11. Were the subjects asked to participate in the study representative of the entire population from which they were recruited?	✓		
	12. Were those subjects who were prepared to participate representative of the entire population from which they were recruited?	✓		
	13. Were the staff, places, and facilities where the patients were treated, representative of the treatment the majority of patients receive?		✓	
**Internal validity**	14. Was an attempt made to blind study subjects to the intervention they have received?			✓
	15. Was an attempt made to blind those measuring the main outcomes of the intervention?	✓		
	16. If any of the results of the study were based on “data dredging”, was this made clear?		✓	
	17. In trials and cohort studies, do the analyses adjust for different lengths of follow-up of patients, or in case-control studies, is the time period between the intervention and outcome the same for cases and controls?	✓		
	18. Were the statistical tests used to assess the main outcomes appropriate?	✓		
	19. Was compliance with the intervention/s reliable?	✓		
	20. Were the main outcome measures used accurate (valid and reliable)?	✓		
**Internal validity confounding**	21. Were the patients in different intervention groups (trials and cohort studies) or were the cases and controls (case-control studies) recruited from the same population?	✓		
	22. Were study subjects in different intervention groups (trials and cohort studies) or were the cases and controls (case-control studies) recruited over the same period of time?		✓	
	23. Were study subjects randomised to intervention groups?	✓		
	24. Was the randomised intervention assignment concealed from both patients and health care staff until recruitment was complete and irrevocable?			✓
	25. Was there adequate adjustment for confounding in the analyses from which the main findings were drawn?	✓		
	26. Were losses of patients to follow-up taken into account?	✓		
**Power**	27. Did the study have sufficient power to detect a clinically important effect where the probability value for a difference being due to chance is less than 5%?	✓		
