# Supplementary material for: Autonomic responses to aerobic and resistance exercise in patients with chronic musculoskeletal pain: A systematic review
Source: PLoS One. 2023 Aug 14;18(8):e0290061. doi: 10.1371/journal.pone.0290061 (PMC10424875; doi:10.1371/journal.pone.0290061)
Supplement: Attachment — Submitted filename: Response_to_Reviewers.docx [file pone.0290061.s004.docx]

December 18, 2022

PLOS ONE

Submission Date: 7 July, 2022

Dear editors and reviewers,

Thank you for inviting us to submit a revised version of our manuscript entitled, “Autonomic responses to aerobic and resistance exercise in patients with chronic musculoskeletal pain: a systematic review” to PLOS ONE (Manuscript Number: PONE-D-22-21106). We also appreciate the time and effort you and each of the reviewers have dedicated to providing constructive and thoughtful feedback on ways to strengthen our draft. Thus, it is with great pleasure that we resubmit our article for further consideration. We have incorporated the revisions reflecting your detailed comments and suggestions. We also hope that the revisions and responses provided below satisfactorily address all the issues and concerns you and the reviewers have noted.

To facilitate your review of our revisions, the following is a point-by-point response to the questions and comments provided in the decision letter dated 11 November, 2022.

Editors’ suggestions:

1. Please ensure that your manuscript meets PLOS ONE's style requirements, including those for file naming. The PLOS ONE style templates can be found at

https://journals.plos.org/plosone/s/file?id=wjVg/PLOSOne_formatting_sample_main_body.pdf and https://journals.plos.org/plosone/s/file?id=ba62/PLOSOne_formatting_sample_title_authors_affiliations.pdf

- We greatly appreciate your time and effort to review our manuscript. Our manuscript has been checked and meets PLOS ONE's style requirements

2. We note that you have stated that you will provide repository information for your data at acceptance. Should your manuscript be accepted for publication, we will hold it until you provide the relevant accession numbers or DOIs necessary to access your data. If you wish to make changes to your Data Availability statement, please describe these changes in your cover letter and we will update your Data Availability statement to reflect the information you provide.

- Thank you for your valuable comment. We do not desire to make any changes to the Data Availability statement.

Reviewer 1 comments:

1. I think the objective of a systematic review must be specific. Introduction needs more specific statements of the question the review addresses. I think it would be better to condense the sentences. Line 83-91 in page 5, needs to be condensed.

- Thank you for your valuable comments. We completely agree with your comments concerning the Introduction section. We have condensed the overall Introduction section, and Lines 78-84, the aim, and research questions of this study have also been condensed for the understanding of the readers. The second and third paragraphs of the Introduction section have been revised to include more specific and logical statements in accordance with the aim of this study (line 52-62, 67-77).

2. In the method section, I think it will be more important to select one of the main outcome among various outcome measures for the autonomic nervous system. Some of the text may be eliminated by the use of Table 2. Table3 can be stated as an Appendix.
   - We greatly appreciate your pertinent comment. If we had to select one of the main outcomes among the different measures, we would choose heart rate variability since this measurement is a more common measurement compared to other autonomic measurements. However, since the topic concerning autonomic activities in exercise in patients with chronic musculoskeletal pain was not sufficiently focused in academic research, only a single value could result in insufficient results. In fact, heart rate variability was utilized in 11/17 articles (64%), indicating that 36% of the included articles would be missed if we selected them as one of the main articles. Additionally, a diversity of the outcome measures for the autonomic nervous system is the innovation and originality of our manuscript. We believe that the insights concerning different autonomic measures would aid clinicians and therapists in the measurement of autonomic dysfunction in clinical practice.
   - We greatly appreciate your valuable comments concerning Tables 2 and 3. We completely agree with you. We have condensed the PICO/PECOs and eligibility criteria in the Methods section using Table 2 (line 107-117). Furthermore, Table 3 has been stated as an Appendix (Supplementary table 2).
   - We also interchanged the locations of Table 1 and 2 because we considered it a more logical sequence for the easier understanding of the readers.
   - Furthermore, “Data extraction and synthesis” in the Methods section was condensed as much as possible (line 154-159).
3. Some of the included studies and references are out of date.
   - Thank you for your valuable comments. We have checked all the references across our manuscript and changed them if the studies were out of date. However, we have retained some past references since they described basic knowledge, such as anatomy or original methods of the questionnaire. Additionally, the included articles have not been changed since a systematic review generally aims to include all the articles published to date.
4. The first paragraph of discussion prefers to summarize the study findings.

- We thank you for your valuable and thoughtful comments concerning the Discussion. We have summarized the study findings to be more comprehensive (line 317-326).

5. In conclusion, I would prefer the results must have external validity or generalizability and future study planning.

- We greatly appreciate your valuable comments. We completely agree your comment. External validity and future planning of research has been added to the Conclusion section (line 417-428).

Minor comments

1. Line 1 in page 5, It would be better to display the references separately.

- Thank you for your valuable suggestion. We have updated all the references and sentences of the Introduction section; the issue of the separated references has been resolved.

Reviewer 2 comments:

1. The authors have conducted a systematic review of autonomic responses to aerobic and resistance exercise in patients with chronic musculoskeletal pain. This is an important topic and a difficult one to analyze. There is enormous heterogeneity in the available publications, in terms of study designs, composition of patient groups, interventions, and choice among derived autonomic measures. The writing style is generally clear and the description of their approach to data retrieval, extraction and analysis is very transparent.
   - We greatly appreciate your constructive and beneficial feedbacks. We have separated your comments for a point-by-point response.
2. To this reviewer, it would make it even more clear to change the language around when one-time exposure to aerobic or resistance exercise is used to probe autonomic responses versus prolonged use of aerobic or resistance exercise over many months as a therapeutic intervention.
   - Thank you for your valuable suggestion. We completely agree with you. We have changed the language concerning one-time exposure and therapeutic intervention in the Introduction (line 78-84), Results (line 238, 280), and Discussion (line 317-326, 328, 361) sections.
3. The authors make a strong effort to analyze the impact of exercise as a therapeutic intervention, and noted that some studies showed improvements in certain measures, while others did not. I would have liked to see more analysis about reasons for success or failure of a therapeutic intervention study. Can the authors find any indication of a dose/intensity/duration effect? As a thought experiment, it could it turn out that : treatment A 30 minutes of moderate aerobic exercise 3 times weekly for 12 weeks is too "small of a dose" to normalize autonomic responses, while treatment B. 1 hour of intense aerobic exercise 5 times weekly and 1 hour of intensive resistance exercise 4 times weekly, both for 12 weeks, does produce clinically significant improvements in derived measures of autonomic function.
   - Thank you for your insightful comments. We have added subtitles in the Discussion section, such as “One time Exposure to aerobic and resistance exercise” or “Aerobic and resistance exercise as therapeutic interventions” (line 328, 361, respectively). Moreover, we have gone through the included articles to seek the possible reasons for success or failure of the results. These changes are now better highlighted than the previous manuscript since the discussion became more positive and constructive for the readers. In accordance with these change in the Discussion, the subsection “Effects of therapeutic interventions” of the Results section and Table 4 have also been revised (line 361-386).
4. In many exercise therapeutic intervention studies, the primary aim is to improve function and pain, and normalizing autonomic physiology can be a secondary outcome. It would be worth knowing the extent to which improvement in function or pain was or was not associated with improvement in derived autonomic measures.
   - We greatly appreciate your valuable comments. An association between a reduction of pain and an improvement in the autonomic nervous system function has been added to the Discussion section. These are important insights, which indicated that the current studies did not sufficiently explore this relationship, i.e., we identified future implications of this research interest (line 387-397).
   - However, we sincerely apologize that a relationship between function and autonomic regulation was not discussed because we did not extract the outcome measures for function in accordance with the protocol, although these are important insights as you have mentioned.
5. In my view, the Conclusion should also make a greater distinction between use of exercise as a probe of autonomic function versus repeated exercise as a therapeutic intervention. I also think that the Conclusion should express greater uncertainty regarding the therapeutic impact of an exercise program. Some readers will read the Conclusion as "exercise does nothing to normalize autonomic regulation" rather than "available studies are mixed and inconclusive regarding impact of exercise on autonomic regulation". Perhaps something like the following: Patients with CMP undergoing a one-time exercise challenge showed variable impairments in several indices of autonomic regulation, when compared to healthy controls undergoing a similar exercise challenge. Therapeutic aerobic or resistance exercise programs extending over periods of weeks to months showed inconsistent impact on autonomic indices in patients with CMP. Further studies of patients with CMP are needed to elucidate whether particular features of an exercise program, including frequency, duration, intensity and type of exercise, are associated with clinically relevant normalization of autonomic regulation.
   - We thank you for your valuable comments and the example of Conclusion. We have modified our Conclusion in accordance with the example you provided. Additionally, we added the statement on generalizability and future implication in accordance with the comments of Reviewer 1 (line 417-428).

Other changes

- We have changed shared co-first authorship since Hironobu Uzawa mainly has written this article.
- The results and conclusion of the Abstract section has been revised in accordance with the reviewers’ comments (line 31-41).
- We sincerely apologize for the incorrect information since Supplementary tables 1 and 2 were opposite. We have added table 3 as appendix; therefore, the current supplementary tables are as follows:
  - S1 Table. PRISMA checklist. (DOCX)
  - S2 Table. Search strategy for MEDLINE. (Xcel)
  - S3 Table. Precise results of the quality assessments. (PDF)
- Table 4 has been renumbered as Table 3 since the original Table 3 was revised as Supplementary table 2. Similar to this revision, Tables 5-8 have been renumbered as Tables 4-7, respectively.
- The result of Gavi et al. has been added to Fig 3 since this information was lacking.
- We deleted the sentence from the Acknowledgments concerning the National Library because they requested us not to state it.

CONCLUDING REMARKS: Once again, thank you for providing us the opportunity to strengthen our manuscript based on your valuable comments and suggestions. We have worked hard to incorporate your feedback and hope that these revisions persuade you to accept our submission.

Sincerely,

Hironobu Uzawa

Corresponding Author

Department of Physical Therapy, School of Health Sciences at Narita

International University of Health and Welfare

4-3, kozunomori, Narita, Chiba, Japan

Tel: +81-476-20-7717

Fax: +81-476-20-7716

Email: h.uzawa@iuhw.ac.jp
